# Stabilisation/Solidification of the Zn-Contaminated Loess Silt in View of the Mechanical Properties

**DOI:** 10.3390/ma17246266

**Published:** 2024-12-21

**Authors:** Agnieszka Lal, Joanna Fronczyk

**Affiliations:** 1 Faculty of Civil Engineering and Architecture, Lublin University of Technology, 40 Nadbystrzycka Str., 20-618 Lublin, Poland; 2 Institute of Civil Engineering, Warsaw University of Life Sciences—SGGW, 166 Nowoursynowska Str., 02-787 Warsaw, Poland; joanna_fronczyk@sggw.edu.pl

**Keywords:** stabilisation/solidification, contaminated loess, zinc, unconfined compressive strength

## Abstract

The effectiveness of the stabilisation/solidification process depends upon a number of factors, the most significant of which are the type of binder, contaminants, and soil undergoing treatment. In accordance with the principles of sustainable construction, alternatives to cement are sought after, with the objective of achieving the lowest environmental impact while maintaining a high level of strength and effective binding of the contaminant. In the study of the stabilisation/solidification of zinc-contaminated loess, incinerated sewage sludge fly ash with reactive magnesia was selected as the binder, and the UCS of the mixtures and microstructure was verified after 28 days of treatment. The values obtained were related to the strength of a reference sample and exhibited by S/S products using Portland cement. The findings verified the effectiveness of the selected materials in the S/S process. Following a 28-day treatment with 30 and 45% IFA and MgO in a 2:1 ratio, the samples were classified as a hard subgrade, suitable for civil engineering purposes, due to the UCS values achieved, ranging from 0.52 to 0.9 MPa. Furthermore, a correlation between the UCS values and the water content was identified, and the mineralogical composition of S/S products was determined with the use of the XRD technique.

## 1. Introduction

The concept of sustainable building engineering is predicated upon minimising energy consumption, both during the construction process and throughout the remaining period of the building’s lifespan. The implementation of this assumption is largely contingent upon the selection of materials that, while meeting the requisite criteria pertaining to the ultimate and serviceability limit states of the structure, have the least possible impact on the environment [1]. Accordingly, the objective in construction processes is to substitute a maximum amount of cement with alternative binders, particularly by-products derived from diverse industrial sources [2]. This initiative aims to mitigate the environmental impact, which is defined here as the carbon footprint of human activities [3]. Furthermore, this trend is also evident in the stabilisation/solidification (S/S) of soils contaminated with heavy metals. In this context, the search for materials other than cement [4] capable of immobilising harmful elements [5] while simultaneously enhancing the mechanical properties of the subsoil [6] is a prominent area of research.

The process of stabilisation/solidification represents a highly time- and cost-effective approach to soil remediation [7], resulting in a continued increase in interest in this method. Improvements to this method are being made in parallel with research into the development of Microbial-Induced Calcite Precipitation (MICP) [8,9], Enzyme-Induced Calcite Precipitation (EICP) [10], and biopolymer treatments (BPTs) [11]. However, the MICP, EICP, and BPT methods are constrained by significant limitations in terms of their applicability. This is due to the high cost of enzymes, difficulties in achieving homogeneous treatment results in large volumes of soil, and challenges associated with soil type and environmental conditions [12]. In an age of intensive urbanisation and the exploitation of increasingly challenging areas (including those contaminated) for construction purposes, the stabilisation/solidification method is becoming an indispensable instrument. In light of the necessity to reduce the application of cement [13], there is a clear necessity for the presentation of research findings which document the efficacy of an alternative binding agents. It is of particular significance to highlight that the effectiveness of the process is determined by three variables: the type of soil, the type of heavy metal contaminant, and the type of binder [14]. Each of these factors is of equal importance; therefore, the selection of an appropriate fixing agent must be adapted to both the type of soil and the type of contaminant, with careful consideration of the findings of reliable studies. A review of the literature data [15] indicates that studies rarely address the issue of loess silt in the context of heavy metal contamination [16,17,18,19,20], particularly in comparison to the numerous publications on S/S sands [21,22,23,24,25,26,27] and clays [28,29,30,31,32,33,34,35,36,37], which limits the range of potential binder alternatives to cement. Heavy metals enter soils from many sources, such as traffic, agricultural and industrial chemical use, and the natural weathering of materials [38,39]. Harmful concentrations of these elements can occur in any soil type, including loess, which forms a considerable part of the world’s land cover.

In view of the lack of sufficient studies for heavy metal-contaminated loess using waste materials and additives to improve the stabilisation/solidification process, it was decided that further research in this area was required.

One study has investigated the stabilisation/solidification of zinc-contaminated loess. This is a highly mobile element [40], and in concentrated quantities, it harms human health [41]. The release of zinc into the environment occurs through a number of processes that are closely related to economic activity, including mining, metallurgy, and wastewater management [42]. Consequently, this represents a significant challenge for engineers in a multitude of contexts, particularly in the development of brownfield sites.

In the context of loess soil and heavy metal contamination (zinc), selecting an appropriate ecological binder for the S/S process remains a key consideration. In the results reported thus far on loess contaminated with zinc, copper, aluminium, and iron [16], fixing agents in the form of class C and F fly ash and slag were used for stabilisation/solidification. In all cases, the addition of type I/II cement was found to be beneficial. In the case of loess containing considerable quantities of As, Cd, Cr, and PB, alternative binders in the form of fly ash (type F), blast furnace slag, lime, and silica fume were employed in the S/S process, in addition to PC [17]. These studies concentrated on the efficacy of the additives employed for binding the contaminants, with minimal attention paid to the strength aspect of the S/S product. In the study conducted by Akhter et al. [17], unconfined compressive strength was not evaluated. It was determined that mixtures with a shear strength, as determined by a pocket penetrometer, greater than 0.35 MPa would be considered acceptable for the further analysis of leaching behaviour. Mahedi et al. [16] refer to UCS values obtained for expansive soils stabilised with type I/II cement and slag [43]. However, it is notable that the UCS values for all mixtures used in the process of stabilisation/solidification [16] have not been subjected to verification. Neither of the papers [16,17] included an analysis of the mineralogical composition prior to and following the S/S process, employing any of the current methods. This was due to the primary objective of the cited works being to ascertain the leaching behaviour of the stabilised loess.

Similarly, in the context of the weathered loess studied by Wei et al. [44], the primary focus was on the leaching of harmful elements. In this instance, the contaminated soil was subjected to stabilisation/solidification using a sustainable phosphate-based binder, and additionally a carbonation process was employed. This study was extended to characterise the leaching behaviour after S/S processes performed simultaneously with acid rain exposure. In a study conducted by Ma et al. on loess solidified with steel slag, calcium carbide slag, and metakaolin [45], the characteristics of micro cracks were established. High-speed photography technology enabled the capture of features associated with the development of fractures. Despite containing information on a novel binder with an environmentally friendly composition, this work does not address the question of the unconfined compressive strength of mixtures with loess. Nevertheless, data on the subject can be found in the work of Xue et al. [46], in which loess was treated with a binder in the form of a composite material comprising a blend of slag, white mud, and calcium carbide residues. It shousld be noted, however, that no contaminant was involved in the studies in [45,46].

Further analysis of the literature data on contaminated sandy and clayey soils has highlighted the potential of using fly ash, a waste product from thermal power plants (FAC) [47], incinerated sewage sludge fly ash (IFA) [48], and an activator in the form of reactive magnesia (MgO) [49,50] with the complete abandonment of cement. Fly ash, an industrial by-product, has been identified as a highly environmentally friendly binder. Reactive magnesia, produced through either dry or wet methods [51], has been established as an acceptable additive when its carbon footprint is demonstrably lower than that of cement while exhibiting the same efficiency as cement in the S/S process. It is estimated that the net amount of CO_2_ entering the environment is 73% lower than during PC production [52], despite the relatively large amount of carbon released during the decomposition of magnesite. This is attributable to the markedly superior carbonation capacity of MgO-based cement in comparison to lime-based cement, as exemplified by PC [53].

The reactive magnesia used as an activator of cementation processes has been observed to produce stable forms of carbonates in the following structures: brucite, nesquehonite, hydromagnesite, and dypingite [53], as confirmed by X-ray diffraction studies. This demonstrates the ability of the material to sequester CO_2_, and furthermore improves the mechanical properties of the S/S product [54]. Fly ash exhibits pozzolanic properties due to its substantial SiO_2_ content in conjunction with Fe_2_O_3_ for IFA [20], Al_2_O_3_ for class F fly ash [55], or CaO for FAC class C fly ash [37].

Given the deficiency of available test data for S/S products of zinc-contaminated loess treated with the specified additives, a preliminary study was conducted [56]. The products of the S/S process were investigated, with the binder consisting of a combination of cement, fly ash, incinerated sewage sludge fly ash, and reactive magnesia. The binder constituted 30% of the dry weight of the soil mixture. The findings demonstrated that substituting two-thirds of the cement with fly ash yielded high UCS results, approximately 16% lower than a mixture treated with cement alone. The combination of fly ash with magnesia proved to be one of the least effective mixtures. Conversely, the beneficial effect on the mechanical properties of the soil after treatment with IFA and an activator in the form of MgO was observed.

The present study aimed to optimise the amount of binder in the form of IFA with the addition of magnesia for the strength of zinc-contaminated loess after S/S treatment. The objective of this study was to ascertain the optimal dosage of the specified green binder, thereby enabling the S/S product to be utilised as a subsoil for foundation or roads subgrade material. In order to achieve the aforementioned objective, it was necessary to design a series of mixes in which the only variable would be the binder content, while maintaining a constant water content and concentration of the selected heavy metal across all mixes with particular attention paid to maintaining the consistent bulk density of all the samples. For comparison, the additional mixes were designed with cement alone and in combination with IFA as the curing agent. The research plan included tests of unconfined compressive strength, which is the significant criterion for evaluating the efficacy of the S/S process, as well as tests of the mineralogical composition of its products. Additionally, an analysis of the water content after the curing period and of the crack patterns formed during the UCS test was planned to complement the aforementioned studies.

## 2. Materials and Methods

### 2.1. Materials’ and Samples’ Preparations

The initial material upon which this study was based was loess, originating in south-eastern Poland, specifically from the Lublin area. This area is geologically part of the Nałęczów Plateau within the Lublin Upland, where loess of various phases can be found. These were formed with the participation of many syngenetic and epigenetic processes [57]. The soil selected for analysis is an aeolian formation, typical of the Late Vistulian Glaciation period. The soil sample was taken from the test plot, dried, and ground to obtain loess silt with a grain size consistent with the natural grain size in the subsoil. The results of the elementary laboratory tests are presented in Table 1.

In light of these findings, the soil was classified as silt (ML) in accordance with the Unified Soil Classification System [59]. The accepted heavy metal was supplied in the form of a suspension of zinc chloride (high-purity, anhydrous) in distilled water at a rate of 2 g Zn^2+^ per 1 kg of soil. This ratio was maintained for all samples.

In light of the findings presented in [56], the current research was designed to optimise the content of green binder combined with an activator in the mixtures. The binder chosen for this purpose was incinerated sewage sludge fly ash (IFA) from the Kraków wastewater treatment plant, while the activator was reactive magnesia of the declared purity of 86%. The binder-to-activator weight ratio was maintained at 2:1 in all mix formulations for this study. The total mass of additives relative to the mass of the mix in combination with the dried soil was adopted in individual samples at levels of 15, 30, and 45%, respectively. In order to ascertain the impact of the activator on the unconfined compressive strength of the S/S product, an additional mixture that did not include the activator was prepared at a ratio of 15% by weight of the dry soil mixture. Furthermore, the intention was to compare the results obtained with samples using a commonly used binder, typically Portland cement (CEMI, class 42.5R), at 15% and cement combined with IFA at a weight ratio of 1:2, which together accounted for 30% of the dry weight of the soil mixture. The percentages of component weights in the dry mixes of the aforementioned compositions, including the symbols assigned to each formula, are presented in Table 2.

All raw materials submitted for analysis were subjected to X-ray fluorescence spectroscopy (XRF), which enabled the determination of their oxide composition. The results obtained are summarised in Table 3.

**Figure 1:**
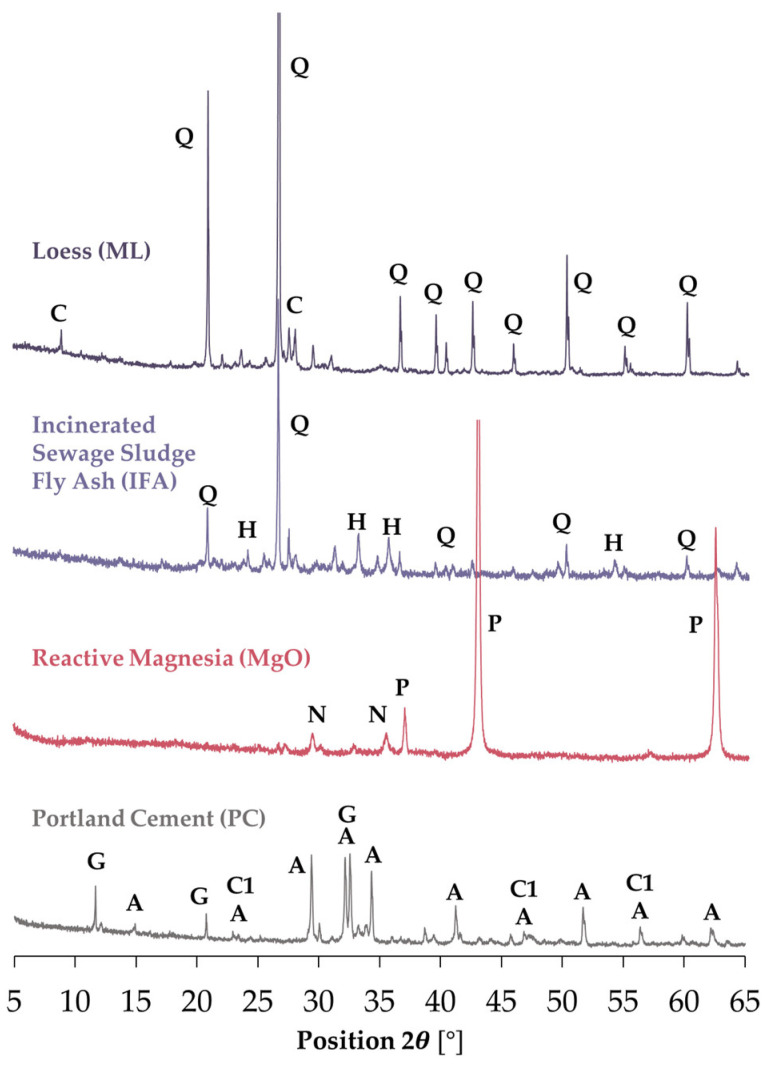
XRD patterns of raw material samples. Symbol used: C—carbon nitrate (C_3_N_4_), Q—quartz (SiO_2_), H—hematite (Fe_2_O_3_), N—sodium nitrate (NaNO_3_), P—periclase (MgO), G—gypsum (CaSO_4_·2H_2_O), A—alite (Ca_3_SiO_5_), C1—calcite (CaCO_3_).

Furthermore, the structure of the applied raw materials was analysed using powder diffraction (XRD) in order to determine the content of individual minerals. The results obtained are presented in Figure 1.

The examination of the loess sample revealed the greatest reflections attributable to quartz, followed by carbon nitrate. The interplanar distances d_hkl_ = 4.25, 3.34, 1.82 Å were identified for quartz, while carbon nitrate exhibited d_hkl_ = 6.84, 3.25, 3.20 Å. Additionally, the analysis revealed the presence of albite, dolomite, and periclase within the loess structure; however, their images were obscured by the intense reflection of SiO_2_. Albite (plagioclase feldspar, NaAlSi_3_O_8_) was identified within the 2θ range of 13.8 to 51.3, with the main d_hkl_ values determined as 6.37, 4.03, 3.18, and 1.78 Å. Dolomite (CaMg(CO_3_)_2_), which belongs to the carbonate cluster and periclase (MgO) from the halite group, was detected relatively infrequently in the spectrum. For dolomite, the interplanar distances were 2.89 and 1.80 Å, while for periclase, d_hkl_ = 2.13 and 1.50 Å. The phase composition was consistent with the findings reported in the world literature [61,62] and in alignment with the X-ray fluorescence test results.

The mineral composition of incinerated sewage sludge fly ash (IFA) has been observed to vary [63]. The IFA sample was found to exhibit intense peaks identifying the presence of quartz (d_hkl_ = 4.26 Å, 3.34 Å, 1.82 Å) and hematite (d_hkl_ = 4.26 Å, 3.34 Å, 1.82 Å). Furthermore, the structure evidenced the presence of calcium iron phosphate, with a chemical formula of Ca_9_Fe(PO_4_)_7_, at reflections d_hkl_ = 3.18 Å, 2.86 Å, 2.59 Å, and chromium titanium antimony oxide (CrTiSbO_6_) at d_hkl_ = 3.25 Å, 1.70 Å.

The sample of reactive magnesia was revealed to contain periclase, which displayed reflections with characteristic interplanar distances of d_hkl_ = 2.11 Å, 1.49 Å. Furthermore, the sample was confirmed to comprise nickel zinc iron oxide (d_hkl_ = 2.53 Å, 1.48 Å) and sodium nitrate (d_hkl_ = 3.04 Å, 2.31 Å).

The list of raw materials used in this study is completed by Portland cement, whose microstructure was composed of gypsum, alite, and calcite. The aforementioned compounds were identified based on the following interplanar distances: d_hkl_ = 7.59 Å, 4.27 Å, 3.26 Å (gypsum), d_hkl_ = 3.04 Å, 2.78 Å (alite), and d_hkl_ = 3.04 Å, 1.88 Å (calcite).

A constant water content was assumed for all sample types. This represents one of the approaches employed in previous studies [64], in contrast to the assumption of an optimal moisture content for each mixture, which is determined individually in a Proctor apparatus [65]. In the present study, the weight of distilled water was measured in order to achieve a moisture content of 15% (±1%) in the contaminated soil sample, inclusive of the weight of additives.

The dried and ground soil was mixed with the binder and activator according to the weight of the ingredients determined for each formulation until a homogeneous mixture was achieved. In the next step, a suspension of zinc chloride in distilled water was inserted and mixed again to obtain a homogeneous water content throughout the volume.

**Figure 2:**
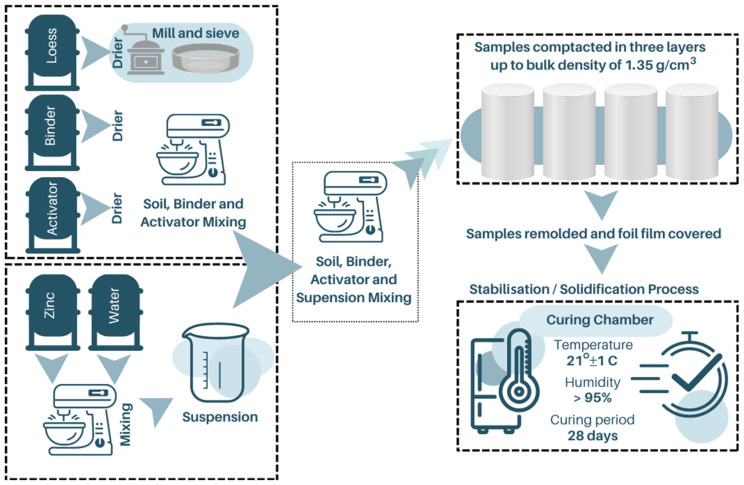
Sample preparation up to stabilisation/solidification procedure.

The formation of the sample required the placement of a specified mass of the mixture in a cylindrical mould with a diameter of 38 mm and a height of 76 mm. The resulting density of the sample was 1.55 g/cm³, which equates to a bulk density of the soil skeleton of 1.35 g/cm³. The stabilised soil was placed in three equal layers, ensuring uniformity across the entire sample. This was achieved by incising the surface of the previously placed layer before compacting the subsequent layer. Samples were prepared in at least four replicates and immediately covered with foil film after removal from the mould. They were then placed in a curing chamber with a constant temperature (21 ± 1 °C) and humidity (>95%) for the assumed 28-day treatment time. A schematic illustration of the sample preparation process is presented in Figure 2.

During the sampling procedure, it was observed that the mixture containing a binder and activator in a 45% ratio of total dry weight was exhibiting difficulties in achieving the required degree of compaction. Instead of gently placing successive layers of the mixture using a wooden cylinder of an appropriate size for the internal dimensions of the mould, it was necessary to apply a significantly higher force and extend the compaction time. The specimens were produced manually, which allowed for the observation of the material’s susceptibility to compaction. It was therefore decided to adopt an additional research formulation, beyond originally planned, in the form of an IFA_M45 mixture with the water content increasing from 15 to 20%, thus obtaining a material with enhanced compactability. The mixture is denoted as IFA_M45_W.

The initial assessment of the water content was conducted on the material remaining after the samples of each mixture were prepared. The subsequent analysis was performed on the samples subjected to unconfined compression testing, which was conducted after a 28-day curing period.

### 2.2. Test Methods

**Figure 3:**
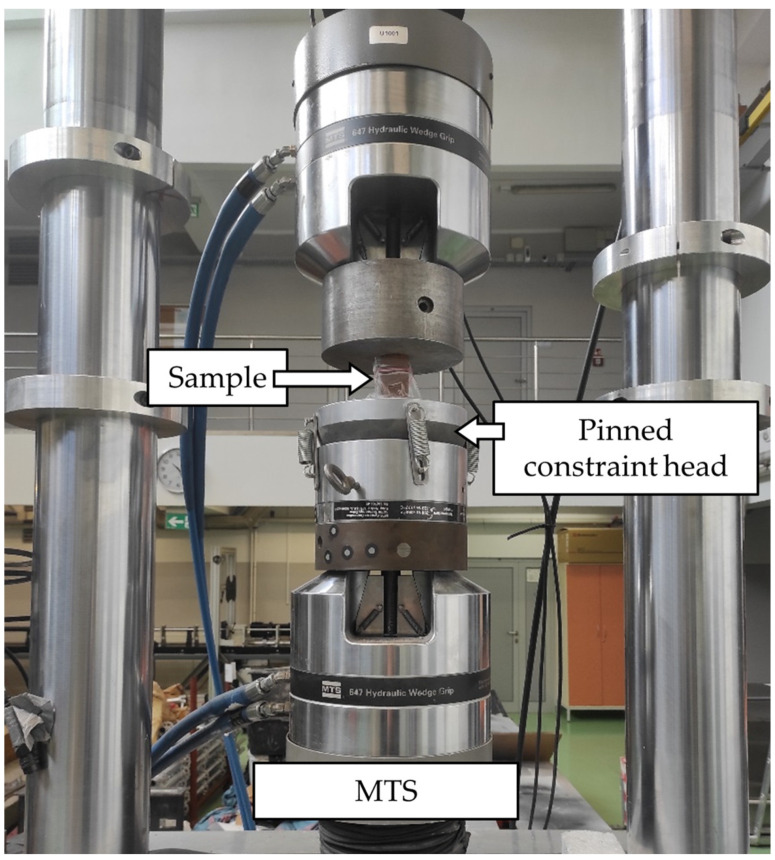
UCS testing equipment.

The test rig employed to determine unconfined compressive strength is based on the MTS 809 Axial/Torsional Test System (MTS, Eden Prairie, MN, USA), with data acquisition in the form of Excel version 2013 files. The instrument is equipped with a pinned constraint head, providing the ability to adjust the specimen surface. The instrument and its associated components are illustrated in Figure 3.

The test was conducted 30 min after the samples were removed from the curing chamber and foil film. The assumed compression rate was 0.2 mm per minute, which corresponded to an axial specimen strain of 0.263% per minute.

An examination was conducted on at least four samples for each designed mixture. Additionally, photographic documentation was conducted on each sample after the demonstration of compression failure, thus allowing for further analysis of the tested mixtures. Immediately following the completion of the testing procedure, each sample was protected from drying for the subsequent determination of the water content for all mixes after a 28-day S/S process.

The strength tests were also accompanied by mineralogical composition analyses of the S/S products. As with the raw materials, the S/S products were analysed with an X-ray diffractometer, the Panalytical X’pert APD, and a PW 3020 goniometer. Additional employed instrumentation included a Cu lamp and a graphite monochromator. All equipment parts are delivered by Malvern Panalytical (Almelo, The Netherlands). In accordance with the established parameters, an excitation energy of 40 kV and 30 mA, a step size of 0.02°, and a scan speed of 0.02° per 2 s were adopted. The samples of each mixture were examined within the range of 5–65°, and the process of determining mineral patterns was supported by HighScore software (version 3.0.5) with the implemented PDF-2 release 2010 database, formalised by JCPDS-ICDD.

## 3. Results and Discussion

### 3.1. Unconfined Compressive Strength

**Figure 4:**
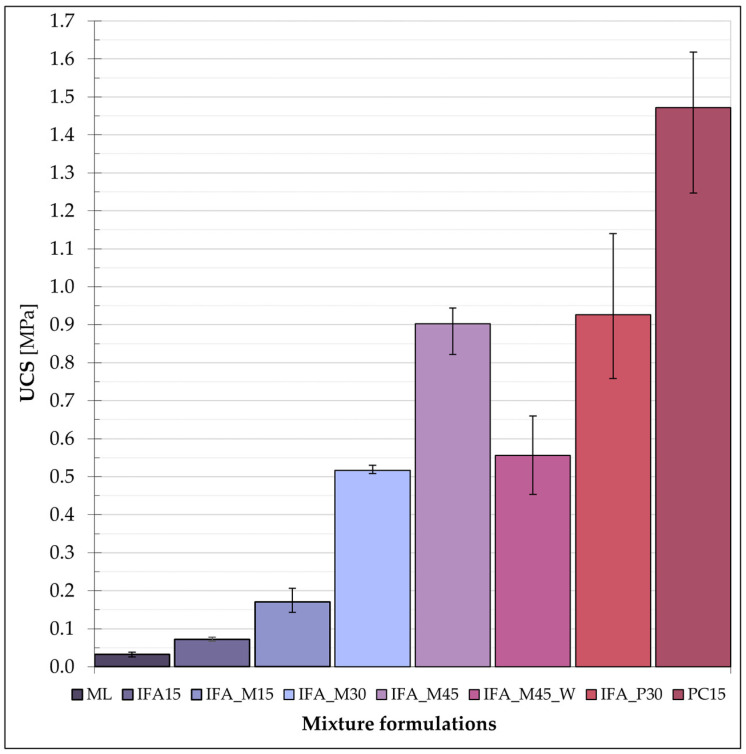
Average values of the unconfined compressive strength of the S/S products.

The unconfined compression strength of the designed formulations was determined through the implementation of strength tests conducted on the planned test rig. The maximum and mean strength values obtained from the tests are presented in Figure 4.

In mixtures exhibiting a strength of less than 0.5 MPa, the standard deviation of the UCS was observed to range between 0.01 and 0.03. In contrast, samples with UCS values exceeding 0.5 MPa demonstrated a notable increase in the range of observed results, with a standard deviation extending from 0.07 to 0.19.

The analysis of the results confirmed the quality of the samples tested and allowed for the conclusion that a homogeneous sample set was obtained, thereby establishing the conformity of the results. Each formulation resulted in a notable improvement in the mechanical properties of the zinc-contaminated loess after the curing process. It is essential to compare the values obtained with the criteria used for stabilised soils, taking into account the intended use of the material in accordance with the relevant standards. The potential application of the S/S product is as a subgrade for road construction. Given the low clay content (particles less than 0.002 mm), the stabilised/solidified loess can be considered non-expansive and, therefore, suitable for this purpose, provided the requisite strength is achieved. Consequently, the UCS values obtained should be compared with the limit values for classifying the subgrade quality [66], as summarised in Table 4.

In the context of employing the S/S product as a building subsoil, it is imperative to simultaneously consider the issue of strength in conjunction with the intensity of the loads that will be supported and the surface area of the foundation. In the context of medium-sized constructions in the single-family housing sector, a value of 0.5 MPa can be considered an appropriate approximation for the stress transferred to the subsoil. The final criterion is the limit value for materials that can be landfilled after treatment [67], which is 0.35 MPa after a 28-day curing time.

The results were analysed in relation to the aforementioned criteria, with a comparative assessment conducted between the tested mixtures. The least effective of the materials employed was incinerated sewage sludge fly ash (IFA), applied as a sole binder at a ratio of 15% by weight to the dry mixture with contaminated soil.

**Figure 5:**
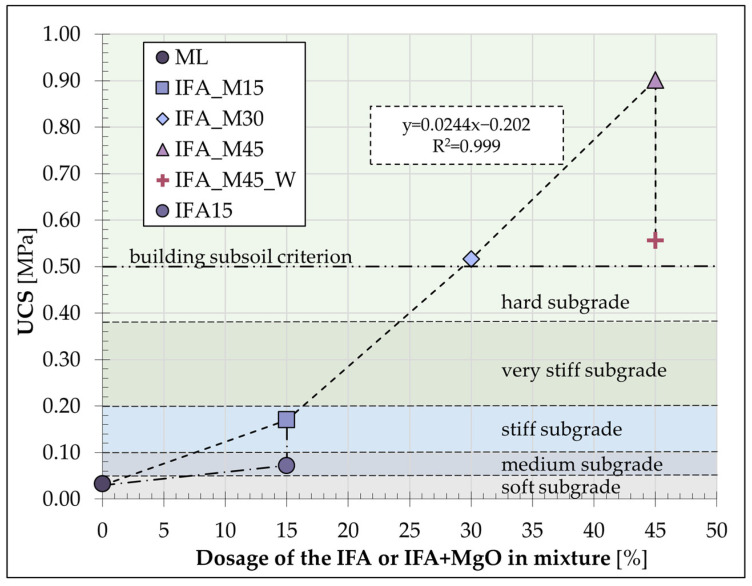
Increase in S/S product strength for mixtures with IFA.

The unconfined compressive strength of the IFA15 mixes was observed to increase to 72 kPa, with the average strength of the untreated loess reported at 33 kPa. The introduction of an activator in the form of MgO, replacing one-third of the IFA weight, led to a significant increase in the strength of the S/S product, exceeding a doubling of the original value. The addition of reactive magnesia to IFA was thus confirmed to be an effective method of the remediation of contaminated loess in terms of strength. The average unconfined compressive strength (UCS) achieved for this mixture is slightly less than 200 kPa. The addition of 15% of the selected additives to the loess resulted in an improvement in the mechanical behaviour from a soft to a stiff subgrade, as illustrated in Figure 5.

It has been demonstrated that the addition of IFA and MgO to a soil mixture in a 30% dry weight ratio results in the achievement of an average compressive strength of 0.52 MPa. The S/S product classification thus corresponds to the hard subgrade range and additionally fulfils the subgrade bearing capacity criterion for medium stresses arising in the soil in single-family housing. An additional increase in the quantity of the IFA and MgO additive incorporated into the contaminated loess to 45% yielded a compressive strength of 900 kPa. This value is comparable to the strength exhibited by the S/S product, which used IFA in combination with Portland cement (in a 2:1 weight ratio), with 30% by weight of these additives in the dry soil mixture. A linear relationship between the mean strength results and binder dosage with a coefficient of determination (R^2^) of 0.999 is evident when considering the UCS results for samples cured with the use of reactive magnesia and incinerated sewage sludge fly ash. This is in accordance with the predictions and findings documented in the literature, which indicate that an increase in the UCS results is associated with an increase in the binder content [68].

The IFA_M45_W formulation was included in the extended test plan due to its low compactability when prepared with a water content of 15%. An increase in the water content of the mixture to 20% resulted in improved compactability; however, the strength test results indicated a decrease in the mechanical properties of the (S/S) product. The average strength of the IFA_M45_W mixture was slightly higher than that of the IFA_M30 mixture.

The PC15 mixture, with Portland cement constituting the binder, demonstrated remarkable strength properties, particularly when considering the binder content in relation to the contaminated silt content. A 15% PC dosage resulted in an average UCS value of 1.5 MPa. Nevertheless, the ecological aspect of the issue remains a priority, and thus the secure and efficient disposal of waste materials with reactive magnesia, which obtained relatively high UCS results, represented a potential application of this type of curing agent. It is crucial to highlight that the IFA_M30 and IFA_M45 mixtures satisfied all three criteria established for evaluation. They possessed sufficient strength for secure deposition, demonstrated the capacity to bear average stresses from buildings within single-family residential construction, and exhibited the potential to provide a high-quality subgrade. These types of product applications have been documented in the relevant literature [69].

A direct comparison of the test results obtained with literature data is not feasible due to the absence of reported results for mixtures of the same composition as those analysed in this study. Accordingly, an analysis was conducted that included mixtures exhibiting similarity in the specified characteristics. The selected literature data are summarised in Table 5, showing, in particular, the type of soil, the type of contamination, the binder, the additive used, and the dosage of the components in the mixture, while the elements that are compatible with the mixtures investigated in this paper are shown in bold.

**Figure 6:**
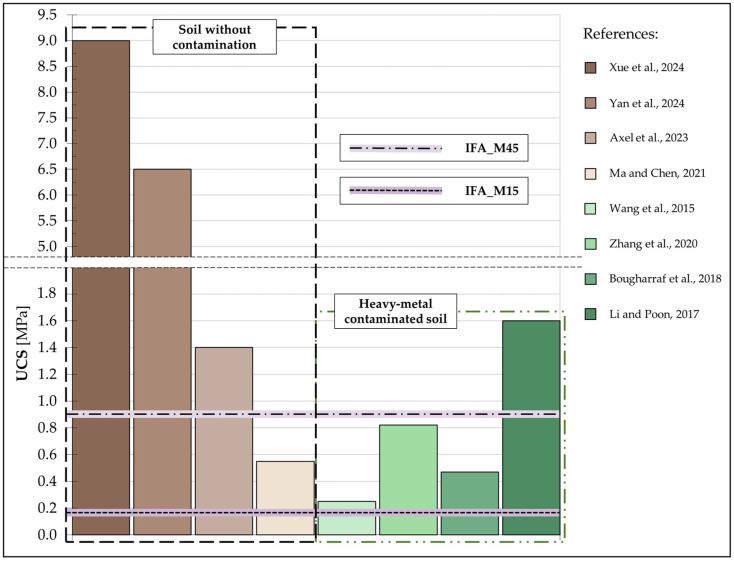
Comparison of the literature UCS values and UCS of the IFA_M45 [46,48,64,68,70,71,72,73].

The UCS values referenced in the cited studies are presented in a summary chart (Figure 6), accompanied by the values obtained for the IFA_M15 and IFA_M45 mixture by the authors of this research.

The results for the unconfined compressive strength (UCS) values achieved using an optimised green binder at a dosage of 45% are competitive with those reported in the existing literature. Nevertheless, direct comparisons are not feasible due to the numerous variables inherent to different studies and the utilisation of disparate equipment.

To highlight this concern, a comparative analysis of the UCS values obtained for contaminated and uncontaminated soils can be used as an illustrative example. The data on the plot indicate that soils stabilised in the absence of contaminants tend to exhibit higher UCS values. Nevertheless, the known test results reveal [74] that the strength of the soil can increase with higher concentrations of contaminants when an appropriate binder is employed. Consequently, any comparisons between different test results must be regarded as approximate. Nevertheless, even preliminary comparisons offer insights into the potential efficacy of the solution under evaluation.

### 3.2. Stress–Strain Behaviour

**Figure 7:**
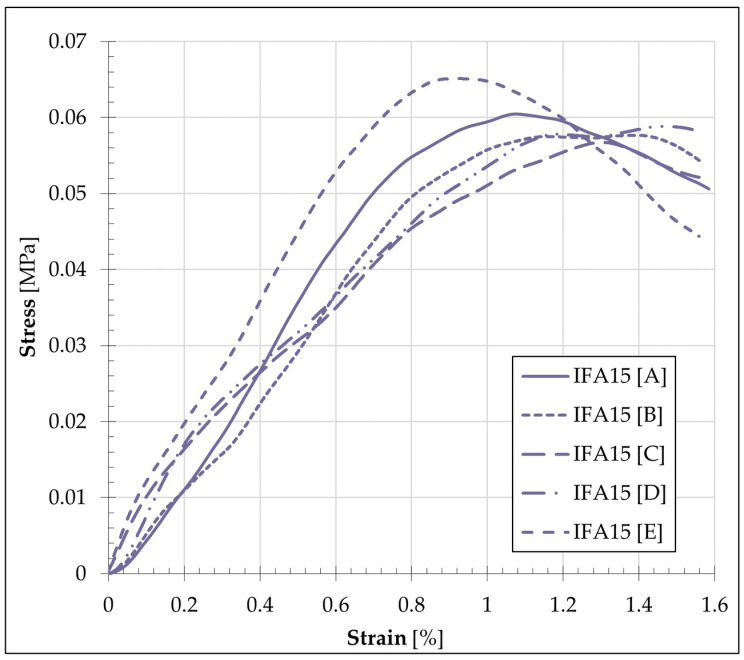
Stress–strain behaviour of the mixture IFA samples.

In addition to measuring the applied load value, the change in specimen height was continuously observed throughout the UCS study. This enabled a comparison of the strain quantity relative to the initial specimen height with the stress value generated during compression. The relationship between strain and stress in the specimen, illustrated using the IFA15 mixture as an exemplar (with graphs representing the data from five specimens labelled A, B, C, D, and E), is presented in Figure 7.

**Figure 8:**
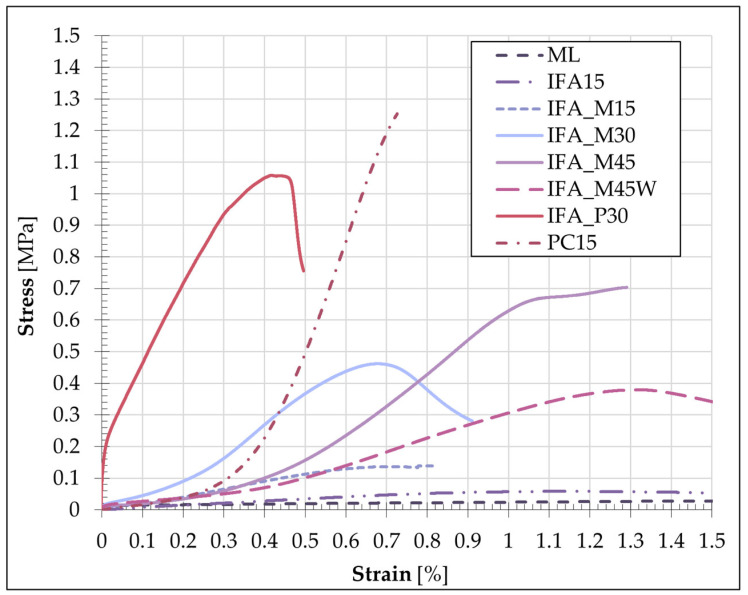
Average values of the stress and strain in each S/S mixture.

The graph of the selected mixture demonstrates that the results obtained for the individual specimens are replicable in terms of both the UCS value and the shape of the stress–strain curve. However, the comparison between all formulation graphs demonstrated notable differences between the mixtures. A summary of the averaged strains and stresses for each of the adopted mixtures is presented in Figure 8.

**Figure 9:**
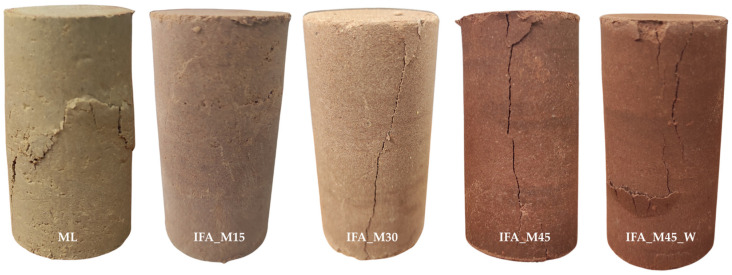
Image of the destruction of selected samples.

Figure 8 supports the findings presented in Section 3.1. The mixes containing cement (PC15 and IFA_P30) demonstrated the highest strength values, as well as the greatest stiffness. The initial stage of testing PC15 reflected the device head’s fitting to the specimen surface without disturbing the overall nature of the deformation. From the graphs, it is possible to identify which mixtures exhibited the characteristics of brittle materials as a result of the stabilisation/solidification process and which remained more deformable. The addition of cement resulted in a notable increase in the binding and hardening of the S/S product. A high level of stress was generated within a narrow range of deformation, after which the occurrence of sample failure was observed. In the case of mixtures containing IFA and reactive magnesia without the addition of cement, the stress increment was less significant, while the specimen was subjected to slightly higher deformations before failure. The soil employed for testing, namely loess silt at the specified water content, exhibited a semi-solid state. Consequently, none of the samples displayed characteristics indicative of plasticity, and failure occurred in each sample no later than at an axial strain of 1%. The failure images of the selected samples, as illustrated in Figure 9, substantiate the aforementioned analyses.

**Figure 10:**
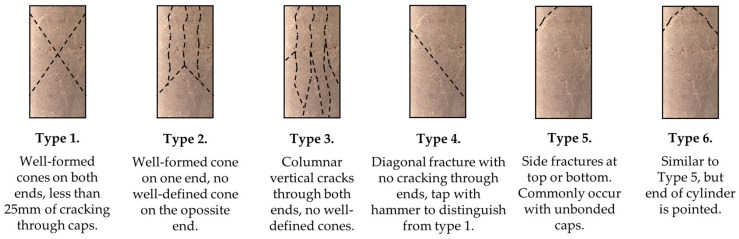
Image of the destruction of selected samples. Figure prepared based on [75].

In specimens exhibiting low strength and relatively increased deformability, the destruction process occurred in an irregular sequence at a height corresponding to approximately half of the specimen. The IFA15, IFA_M15, and soil-free additives (ML) were deformed to the centre of their volume. The damage observed in samples IFA_M30 and IFA_M45 was comparable to that observed in samples IFA_P30 and PC15, which exhibited cracks characteristic of brittle materials. The above crack patterns can be compared to the general behaviour of concrete specimens undergoing compression. In accordance with the schemes proposed by Shuvo et al. [75], Figure 10 illustrates the typical crack patterns observed in cylindrical specimens.

The most durable of the S/S products (IFA_M45, PC15) exhibited vertical cracking (type 3 according to Figure 10), compared to the softer mixtures, which displayed slightly diagonal cracking. The IFA_M45_W specimen exhibited considerably greater deformation than specimens of similar strength but lower water content, accompanied by vertical cracking. Additionally, the surface of the specimen demonstrated the formation of cavities, indicating further damage. The damage observed can be classified as a combination of types 3 and 6 (see Figure 10). These observations, in conjunction with the correlation between material stiffness and these phenomena, indicate a potential relationship with the water content of the tested specimens, which will be discussed in the subsequent section.

### 3.3. Water Content

**Figure 11:**
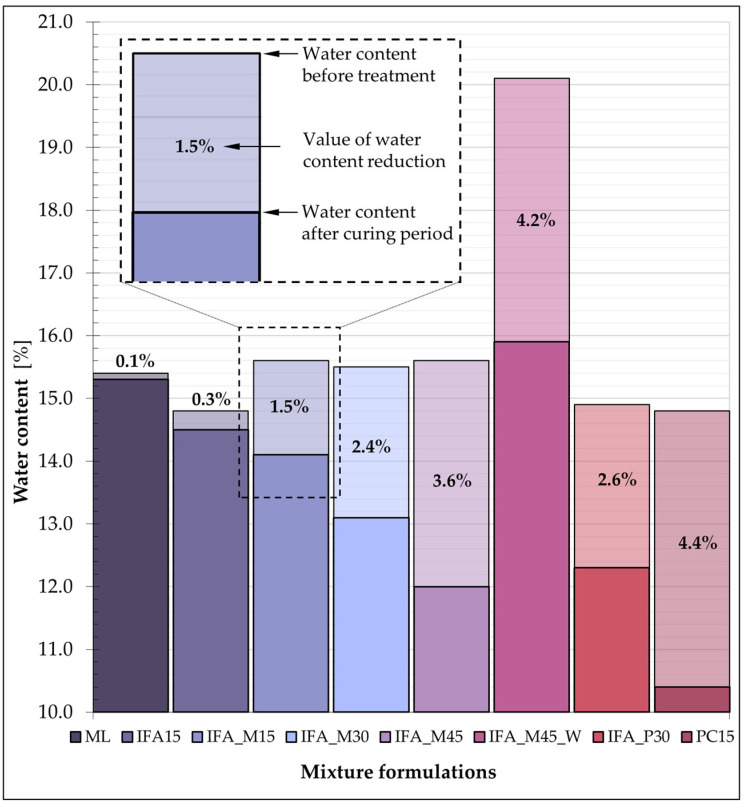
A comparison of the water content at the initial and final stages of the curing period.

The water content of each mixture was analysed at two stages: immediately after the samples were prepared and after stabilisation/solidification, which corresponds to 28 days curing time. The mixtures were found to be highly sensitive to external conditions; therefore, it was imperative to adhere to the test sample preparation procedure and maintain constant conditions throughout the curing process. The determined water content values for each mixture are presented in Figure 11.

**Figure 12:**
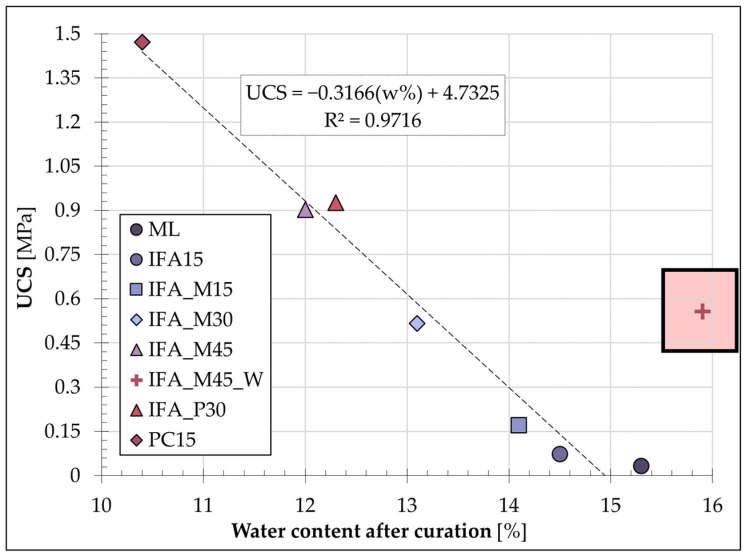
Average water content after the S/S curing period against average UCS values for S/S mixtures.

The values presented are the averages derived from a minimum of four samples for each mixture. The highest average water loss (4.4%) was observed in the comparison mixture, which consisted of soil and Portland cement, and in the IFA_M45_W mixture (4.2%). The IFA_M45 formulation exhibited a slightly reduced water loss (3.6%), while the IFA_M30 (2.4%) and IFA_P30 (2.6%) mixtures demonstrated noticeably lower water loss. The aforementioned comparative analysis, when considered in conjunction with the UCS values determined for the individual blends, suggests the existence of a relationship between the two sets of data. Figure 12 summarises the average strength values of the individual mixes and the water content of the samples immediately following the UCS test.

All mixtures with an initial water content of 15% (±1%) followed a linear trend with a very high coefficient of determination (R^2^ = 0.97), in which greater sample water loss was correlated with increased S/S product strength. The resulting relationship was indicative of the intensity of hydration processes occurring in the mixtures during the 28-day stabilisation/solidification period. This thesis was made possible by the adoption of the procedure in terms of sample preparation and care and was confirmed by the preservation of the initial water content by ML samples containing neither a binder nor additives.

A mixture with an initial water content of 20%, which exhibited a significant divergence from the plotted trend, was excluded from the linear relationship. A comparison was therefore undertaken between samples IFA_M45_W and IFA_M45, which differed only in their initial water content. The strength results obtained for the sample with a water content of 20% were found to be approximately twice as low as those obtained for the mix with an initial water content of 15%. These findings are consistent with the findings of the previously published literature, which indicate that the UCS values achieved are lower when the initial water content of the S/S-treated mixture is higher [76]. The water/binder ratio for the compared samples was 0.33 (IFA_M45) and 0.44 (IFA_M45_W). In both cases, the quantity of water did not result in the mixture achieving a plastic consistency. The nature of the soil (which had a low plasticity index) permitted the unbounded absorption of water by the binder, thereby preventing any interference with the formation of the S/S product strength in either mixture. The reduced strength of the IFA_M45_W mixture can, therefore, be attributed solely to the higher water content of the sample at the time of testing in comparison to the IFA_M45 sample.

**Figure 13:**
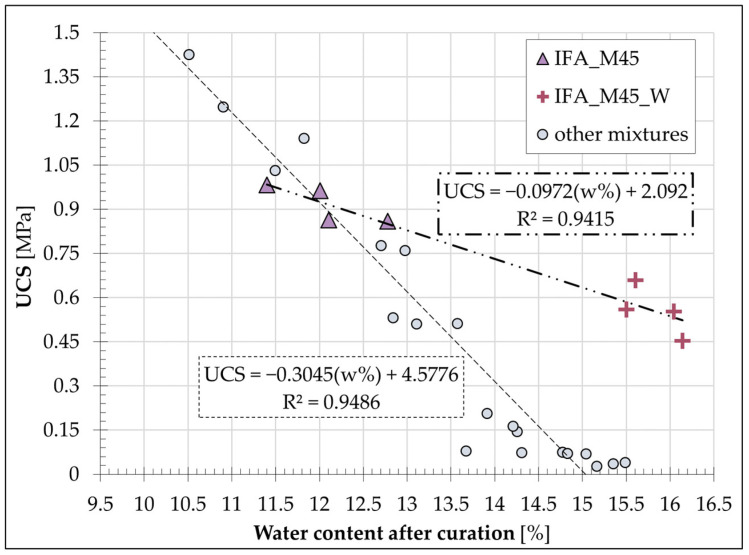
A comprehensive study of the fluctuations in water content observed in the S/S mixtures against obtained UCS values.

In consideration of the aforementioned findings, individual samples from each of the highlighted mixtures were subjected to analysis in comparison to the other formulations, with a particular focus on water content and unconfined compressive strength. The collective data are presented in Figure 13.

It is established that the water content of S/S products had a noticeable impact on the compressive strength of mixtures with an equal dry content. It is significant to note that modifying the initial water content of the IFA_M45 base mixture by 5% (to IFA_M45_W) resulted in a reduction in the observed UCS values by an average of 40%. These values demonstrated relatively close values to the strength exhibited by the IFA_M30 mixture, which contained a binder content that was reduced to two-thirds.

### 3.4. Mineralogical Analysis

**Figure 14:**
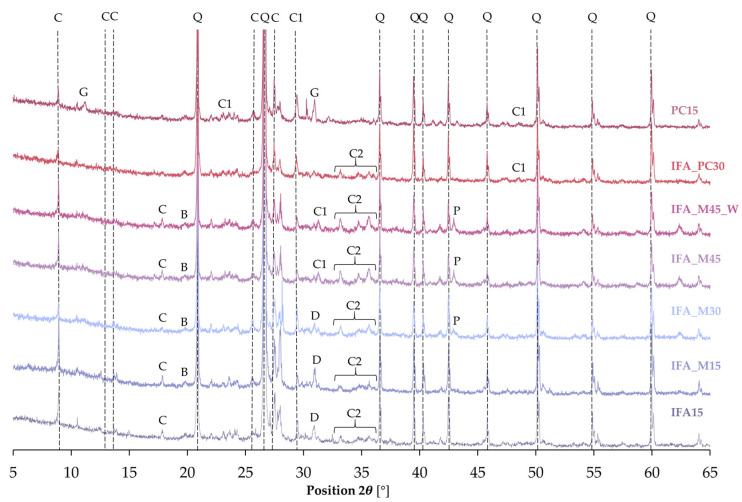
XRD patterns of the S/S product. Symbols used: B—brucite, C—carbon nitrate, C1—calcite, C2—calcium iron phosphate, D—dolomite, G—gypsum, P—periclase, Q—quartz.

Mineralogical studies were conducted using an X-ray diffractometer (Malvern Panalytical, Almelo, The Netherlands) on samples of mixtures that had been dried and suitably pulverised following a 28-day stabilisation/solidification process. The composition of the S/S products was assessed and compared between individual mixtures and in relation to the findings of the raw material testing. Figure 14 illustrates the X-ray diffraction (XRD) patterns of the mixtures after the curing process.

In the above figure, the compounds identified on the basis of the highest-intensity reflections have been marked. The analysed spectra were dominated by peaks corresponding to quartz. This finding is consistent with the results of the XRF oxide composition study, which confirmed this mineral to be the main constituent of the loess. The analysis revealed the presence of carbon nitride in all the samples, which can be attributed to the contribution of this component in the soil structure. Furthermore, dolomite was detected in samples containing up to 30% binder. The aforementioned mineral plays a role in the formation of the silt structure, as evidenced by the XRD test conducted on the raw material. As the proportion of binder increased, the presence of dolomite was no longer observed; however, calcite was identified in the same 2θ position range. Calcite was also noted in mixtures employing cement, while PC15 additionally contained gypsum. The presence of brucite was observed in mixtures with the use of the activator in the form of the reactive magnesia, while mixtures IFA_M30, IFA_M45, and IFA_M45_W also revealed peaks characteristic of periclase. All S/S products containing incinerated sewage sludge as a binder display indications of the presence of calcium iron phosphate, a compound previously observed in the raw material.

The recognised mineralogical compositions of the S/S products of the studied mixtures, including those with lower-intensity peaks, are presented in a summary table (Table 6), which also includes the ideal formula and characteristic interplanar distances [Å]. Furthermore, a literature reference is provided in which the reported experimental results correspond with the present findings.

## 4. Conclusions

The planned research programme was completed with an additional mixture formulation, with the water content increased from 15% to 20%. This extension was caused by difficulties encountered in achieving the desired level of compaction in a mixture comprising a binder with additives, which constituted 45% of the mixture dry mass. The objective of this study, to determine the amount of green binder additive, allowing the S/S product to be used in civil engineering, was achieved. The analysis of the results in terms of unconfined compressive strength, axial deformation, and changes in water content yielded the following conclusions:

(1) The application of each of the curing agents resulted in an increase in the 28-day compressive strength of the contaminated loess in comparison to the reference sample, representing the untreated loess with zinc contamination.
(2) The addition of reactive magnesia (MgO) improved the unconfined compressive strength of the product solidified/stabilised with fly ash derived from incinerated sewage sludge (IFA). The substitution of one-third of the weight of IFA with MgO in mixtures containing 85% loess led to a twofold increase in UCS values.
(3) Raising the dosage of binder and activator content (in a weight ratio of 2:1) in the loess mixture from 15% to 30% and 45% increased the achievable UCS values by an average of 216 and 386 kPa, respectively.
(4) The stabilisation/solidification of zinc-contaminated loess was effectively achieved through the utilisation of IFA and reactive magnesia (in the weight ratio binder to additive assumed as 2:1), resulting in a compressive strength that meets the criteria for a hard subgrade and a subsoil with sufficient bearing capacity for supporting the average stresses occurring in the ground beneath single-family housing structures.
(5) The unconfined compressive strength of the examined mixtures did not exceed that of a comparative mixture containing 15% Portland cement (PC15). However, the satisfactory UCS values and the ecological aspect indicate the potential for successfully utilising IFA and an activator in the form of MgO at 30 and 45% of the dry weight of the mixture to stabilisation/solidification zinc-contaminated loess.
(6) The utilisation of cement as well as an increased proportion of green binder in the mixture had been observed to result in an enhanced unconfined compressive strength of the S/S products. Consequently, the material exhibited a notable increase in brittleness.
(7) The specimens were destroyed at an axial strain that did not exceed 1%.
(8) The reduction in the water content of S/S product in comparison to the initial water content of the mixture is found to be correlated with the achieved unconfined compressive strength. In the mixtures tested, the loss of water is observed to be proportional to the increase in UCS, exhibiting a linear relationship.
(9) An increase in the initial water content of the mixture by 5% at an additive ratio of 45% in the dry matter results in an average reduction of 40% in the achieved UCS values after 28 days of treatment.

These findings underscore the potential of a binder composed of incinerated sewage sludge fly ash and magnesium oxide (MgO) for stabilising zinc-contaminated loess. However, several constraints and challenges associated with the implementation of this solution at contaminated sites must be addressed. A primary concern is the variability of incinerated fly ash (IFA), which lacks a certified composition and properties, leading to fluctuations based on the treatment plant of origin and the temporal aspects of its production. Additionally, the method of introducing the binder into the contaminated site is critical. On a macro scale, achieving a homogeneous mixture of binder components with the soil and ensuring adequate compaction can pose substantial challenges. The effectiveness of the proposed method in mitigating zinc leaching is another significant issue. The efficient retention of zinc within contaminated areas remains a primary challenge, as it directly impacts the long-term stability and safety of the remediation process. Moreover, ageing factors that lead to the deterioration of solidification/stabilisation (S/S) product properties over time are also significant. These factors can undermine the efficacy of the binder in long-term applications. The aforementioned issues are planned to be verified in further research.

## Figures and Tables

**Table 1:** Characteristics of loess silt.

Soil Characteristic	Quantity	Test Methodology
Specific bulk density	2.66 g/cm^3^	PN-EN ISO 17892-1 [58]
Plastic limit PL	20.4%	PN-EN ISO 17892-12 [58]
Liquid limit LL	26.0%	
Grain size distribution:		PN-EN ISO 17892-4 [58]
Clay (<0.002 mm) [%]	2.55	
Silt (0.002–0.063 mm) [%]	82.8	
Sand (>0.063 mm) [%]	14.95	
Soil classification	ML	ASTM D2487 [59]
Natural water content [%]	12	PN-EN ISO 17892-1 [58]
Optimum water content [%]	11.86	ASTM D698 [60]

**Table 2:** Dry components of mixture formulations.

Mixture Formulations Symbol	Dry Components of Mixtures			
	ML [%]	PC [%]	IFA [%]	MgO [%]
ML	100	-	-	
IFA15	85	-	15	-
IFA_M15	85	-	10	5
IFA_M30	70	-	20	10
IFA_M45	55	-	30	15
IFA_M45_W ^1^	55	-	30	15
IFA_P30	70	10	20	-
PC15	85	15	-	-

^1^ Mixture with an initial water content of 20%.

**Table 3:** Oxide composition of raw materials.

Material	Percentage in Weight [%]									
	SiO_2_	Al_2_O_3_	Fe_2_O_3_	CaO	MgO	K_2_O	P_2_O_5_	SO_3_	TiO_2_	LOI ^1^
ML	74.53	8.13	2.85	4.58	1.24	2.80	nd ^2^	nd	0.71	3.45
IFA	31.82	6.35	19.42	12.21	3.15	2.08	18.81	1.60	1.18	2.34
MgO	0.98	0.26	12.14	9.40	72.06	nd	nd	0.60	nd	4.34
PC	15.28	2.93	4.15	68.11	0.38	0.62	nd	3.72	0.28	3.14

^1^ Loss of ignition, ^2^ not detected.

**Table 4:** Subgrade quality classes.

UCS [kPa]	25–50	50–100	100–200	200–380	>380
Subgrade’s quality	soft subgrade	medium subgrade	stiff subgrade	very stiff subgrade	hard subgrade

**Table 5:** Data of the samples adopted for the comparative analysis.

Soil Type	Cont ^1^	Binder	Dosage	Curing Time [Days]	Additional Notes	Reference
**Loess** ^3^	-	slag, white mud, and calcium carbide residues	**15%**	**28**	4 × 4 × 16 mould	Xue et. al [46]
**Loess**	-	solid waste coal gangue and magnesium oxysulfate	6.5%	**28**	sample of 5 cm in height and diameter	Yan et al. [68]
**Loess**	-	cement	3%	**28**	61.8 mm diameter and 125 mm height	Axel et al. [70]
**Loess**	-	lime	25%	**28**	7.07 cm cube samples	Ma and Chen [71]
Sand ^2^	Pb, **Zn**, Cr, As, Cu, Ni, organic	**reactive magnesia**	**15%**	**28**	In situ S/S	Wang et al. [72]
Sediment (silty sand)	Al, Cr, Mn, Fe, Ni, Cu, **Zn**, As, Cd, Ba, Pb	ground granulated blast-furnace slag, Portland cement	12%	**28**	sample of 10 cm in height and 5 cm in diameter	Zhang et al. [73]
Sand-coloured clayey	Cr, Cu, **Zn**, Pb, Cd, Ni	calcium aluminate cement, bentonite	12.8%	**28**	sample of 10 cm in height and 5 cm in diameter	Bougharraf et al. [64]
Silty clay	Pb	Portland cement, **Incinerated** **Sewage sludge fly ash**	17%	**28**	sample of 5 cm in height and diameter	Li and Poon [48]

^1^ Cont.—type of the contaminant; ^2^ with anthropogenic additives; ^3^ bold was used to the elements compatible with the mixtures investigated

**Table 6:** The mineralogical composition of the researched mixtures.

Compound/Mineral Name	Mixture Symbol	The Characteristic Interplanar Distances [Å]	Ideal Formula	Ref ^1^
quartz	all mixtures	4.25, 3.34	SiO_2_	[61]
calcite	mixtures with more than 40% of binder	3.04, 1.88	CaCO_3_	[68]
dolomite	IFA15, IFA_M15, IFA_M30	2.89, 2.40	CaMg(CO_3_)_2_	
carbon nitrate	all mixtures	9.89, 3.25	C_3_N_4_	
calcium iron phosphate	all mixtures with IFA	2.67, 2.54	Ca_9_Fe(PO_4_)_7_	
brucite	mixtures with MgO	4.48, 2.28	Mg(OH)_2_	[51]
gyps	PC15	7.59, 4.28	CaSO_4_·2H_2_O	[53]
periclase	IFA_M30, IFA_M45, IFA_M45_W	2.12, 1.50	MgO	[77]

^1^ Ref—A reference indicating the presence of the mineral/compound.

## Data Availability

The original contributions presented in this study are included in the article. Further inquiries can be directed to the corresponding author.
